# Comparison of Three Consecutive Monthly Administrations Between Aflibercept 8 mg and Brolucizumab 6 mg in Polypoidal Choroidal Vasculopathy

**DOI:** 10.3390/ph18121811

**Published:** 2025-11-27

**Authors:** Yoshiko Fukuda, Yoichi Sakurada, Yumi Kotoda, Misa Kimura, Kenji Kashiwagi

**Affiliations:** Departments of Ophthalmology, Faculty of Medicine, University of Yamanashi, Chuo 409-3821, Yamanashi, Japan; ysugiyama@yamanashi.ac.jp (Y.F.); ykanai@yamanashi.ac.jp (Y.K.); smisa@yamanashi.ac.jp (M.K.); kenjik@yamanashi.ac.jp (K.K.)

**Keywords:** aflibercept 8 mg, brolucizumab, polypoidal choroidal vasculopathy, dry macula, the complete regression rate

## Abstract

**Purpose:** The aim was to compare the short-term outcomes of aflibercept 8 mg and brolucizumab for the treatment of polypoidal choroidal vasculopathy (PCV). **Methods:** This study included 48 eyes of 48 patients with PCV. Drug selection was based on the treatment period. Sixteen eyes received aflibercept 8 mg and thirty-two eyes received brolucizumab. All eyes underwent three consecutive monthly injections: aflibercept (114.3 mg/mL; 0.07 mL) or brolucizumab (120 mg/mL; 0.05 mL). Indocyanine green angiography was performed at baseline and at the 3-month visit to confirm the presence of polypoidal lesions. **Results:** In the aflibercept 8 mg group, best-corrected visual acuity (BCVA) significantly improved from 0.28 ± 0.26 at baseline to 0.18 ± 0.25 at the 3-month visit (*p* < 0.001). In the brolucizumab 6 mg group, BCVA improved from 0.35 ± 0.26 to 0.29 ± 0.27, although the change was not statistically significant (*p* = 0.08). Multivariate regression analysis showed that better BCVA at 3 months was associated with better baseline BCVA and lower central retinal thickness (CRT), independent of the drug used. CRT decreased from 382 ± 157 to 198 ± 98 in the brolucizumab 6 mg group and from 358 ± 152 to 192 ± 76 in the aflibercept 8 mg group at 3 months. Subfoveal choroidal thickness (SCT) decreased from 201 ± 78 to 167 ± 60 in the brolucizumab 6 mg group and from 186 ± 76 to 153 ± 67 in the aflibercept 8 mg group. The dry macula rate at 3 months was the same for aflibercept 8 mg and brolucizumab 6 mg at 93.8%. Complete regression of polypoidal lesions was observed in 62.5% and 75.0% of patients in the aflibercept and brolucizumab groups, respectively (*p* = 0.57). **Conclusions:** During the induction phase, aflibercept 8 mg demonstrated comparable outcomes to brolucizumab 6 mg in reducing CRT and SCT, achieving a dry macula, improving BCVA, and regressing polypoidal lesions in eyes with PCV.

## 1. Introduction

Polypoidal choroidal vasculopathy (PCV), a subtype of type 1 macular neovascularization (MNV) secondary to exudative age-related macular degeneration (AMD), is characterized by polypoidal lesions with or without type 1 MNV on indocyanine green angiography (ICGA) [1]. In some cases, late-phase ICGA reveals hyperfluorescence, a phenomenon known as choroidal vascular hyperpermeability [2,3]. This feature is often observed in eyes with pachychoroid spectrum disorders and is associated with focal or diffuse choroidal thickening, dilated outer choroidal vessels, and attenuation of the inner choroid [4]. In eyes with PCV, soft drusen and reticular pseudodrusen, also known as subretinal drusenoid deposits, are not seen frequently compared with neovascular AMD and retinal angiomatous proliferation; however, pachydrusen are frequently seen around the macular area or along the vessels [5], which is associated with increased choroidal thickness and shows hyperfluorescent spots on late-phase ICGA.

PCV is the most common subtype of advanced AMD among Asians, with a reported prevalence of 40–60% [6,7]. In contrast, its prevalence among Caucasians is significantly lower, accounting for less than 10% of exudative AMD cases [8], although ICGA is not performed routinely in Caucasians.

The TAP study, which evaluated photodynamic therapy (PDT) in 609 patients with exudative AMD, found that while PDT did not improve best-corrected visual acuity (BCVA) in eyes with exudative AMD, it slowed BCVA deterioration compared with placebo controls [9]. In clinical practice, PDT has proven effective in inducing the regression of polypoidal lesions in PCV, along with a reduction in choroidal thickness, and visual outcomes after one year tend to be better than those in neovascular AMD [10]. However, long-term visual decline may still occur due to recurrent exudation, including subretinal hemorrhage and subretinal/intraretinal fluid, and macular atrophy.

Vascular endothelial growth factor (VEGF) is a key mediator of retinal and choroidal vascular diseases, including diabetic retinopathy, retinal vein occlusion, and exudative AMD [11]. To date, several VEGF inhibitors are commercially available.

The VISION study, a randomized clinical trial to evaluate the safety and efficacy of the first VEGF inhibitor, pegaptanib, demonstrated that the mean BCVA gradually decreased in eyes with exudative AMD at 54 weeks despite 6-weekly intravitreal administration of pegaptanib in 1186 eyes with exudative AMD, irrespective of the three concentration types—0.3 mg, 1.0 mg, and 3.0 mg [12]. However, BCVA deterioration is milder in the pegaptanib treatment group than in placebo controls. It was found that pegaptanib did not improve BCVA in eyes with exudative AMD, like PDT. There are five isoforms in VEGF-A. Pegaptanib was designed to selectively inhibit a VEGF-A 165 isoform. It is speculated that the resolution of retinal/intraretinal fluid is insufficient in eyes treated with pegaptanib because of the selective blockage of the VEGF-A 165 isoform. However, the large-scale study was not performed on the treatment of PCV using pegaptanib. This is probably because the response to pegaptanib was poor in eyes with PCV.

Since the introduction of ranibizumab, VEGF inhibitors have become the standard treatment for exudative AMD. The ANCHOR/MARINA trial evaluated the efficacy and safety of monthly dosing of ranibizumab in eyes with exudative AMD [13,14]. The ANCHOR trial investigated whether monthly dosing of ranibizumab was effective for the improvement of BCVA compared with the treatment of PDT in eyes with classic macular neovascularization. The 423 patients were randomly assigned to the ranibizumab group and the PDT group. The ranibizumab group was further subdivided into the 0.3 mg concentration group or the 0.5 mg concentration group. BCVA improved by 15 letters or more in 35.7% of the 0.3 mg group and 40.3% of the 0.5 mg group, as compared with 5.6% of the PDT group. At 12 months, BCVA improved by 8.5 letters in the 0.3 mg group and 11.3 letters in the 0.5 mg group, as compared with a decrease of 9.5 letters in the PDT group. The MARINA trial investigated whether monthly dosing of ranibizumab was effective for the improvement of BCVA in eyes with minimally classic or occult macular neovascularization compared with placebo controls. A total of 716 patients were randomly assigned to the ranibizumab 0.3 mg, the ranibizumab 0.5 mg, and the sham injection group. BCVA improved by 15 letters or more in 24.8% of the ranibizumab 0.3 mg group, 33.8% of the ranibizumab 0.5 mg group, and 5.0% of the sham injection group. The mean BCVA improvement was 6.5 letters in the ranibizumab 0.3 mg group and 7.2 letters in the ranibizumab 0.5 mg group, as compared with a decrease of 10.4 letters in the sham injection group. In both ranibizumab treatment groups, BCVA was maintained throughout the study period. The LAPTOP study compared the efficacy of ranibizumab with PDT in eyes with PCV [15]. Patients were assigned to the ranibizumab arm (46 eyes) or the PDT arm (47 eyes). Patients in the ranibizumab arm received three consecutive monthly administrations of ranibizumab 0.5 mg, while those in the PDT group received one session of PDT at baseline. Additional treatment was performed as needed in each arm. In the PDT group, the mean log MAR BCVA was not changed (0.57 ± 0.31 to 0.60 ± 0.40), whereas in the ranibizumab group, the mean log MAR BCVA significantly improved from 0.48 ± 0.27 to 0.39 ± 0.26. However, central retinal thickness significantly decreased in both groups. To date, there have been no randomized clinical trials reporting the efficacy and safety comparing ranibizumab with PDT in eyes with PCV, except this study.

The VIEW 1 and 2 study evaluated whether bimonthly dosing of aflibercept 2 mg after three consecutive monthly induction phases was comparable to monthly dosing of ranibizumab in terms of BCVA improvement and anatomical response using a total of 2419 patients. Patients were randomly assigned to the monthly aflibercept 0.5 mg group, the monthly aflibercept 2.0 mg group, the bimonthly aflibercept 2.0 mg group, and the monthly ranibizumab 0.5 mg group. All aflibercept groups were noninferior in terms of BCVA improvement and clinically equivalent to the monthly ranibizumab group in terms of anatomical measures. There were no large-scale randomized studies evaluating the efficacy and safety of intravitreal administrations of aflibercept 2.0 mg monotherapy in eyes with PCV. However, PCV also responds well to VEGF inhibitors, such as ranibizumab and aflibercept, both as monotherapy and in combination with PDT. Notably, combination therapy has demonstrated better BCVA improvement in eyes with PCV, which is probably because VEGF inhibitors decrease VEGF levels in the vitreous humor and protect the macular ischemia due to PDT, along with a high rate of polypoidal lesion occlusion.

Brolucizumab, the smallest commercially available VEGF inhibitor by molecular weight (26 kDa), has been approved by the U.S. FDA for use in AMD. It can achieve stability and solubility at high doses in a single intravitreal injection of 50 μm. At a dose of 6.0 mg, the equivalent molecular dose of brolucizumab was approximately 10 times greater than aflibercept 2.0 mg and 20 times greater than ranibizumab [16]. The HAWK and HARRIER trials were designed to compare the efficacy of brolucizumab with aflibercept in eyes with exudative AMD. The patients were randomly assigned to the aflibercept 2.0 mg group, the brolucizumab 3.0 mg group, and the brolucizumab 6.0 mg group. The aflibercept 2.0 mg group received an 8-week interval injection of aflibercept up to 48 weeks after three consecutive monthly intravitreal administrations. The brolucizumab group received three consecutive monthly administrations of brolucizumab. If the macula was dry on SD-OCT at each visit, the treatment interval was maintained for 12 weeks. However, once exudation was seen on OCT, the treatment interval was shortened to 8 weeks. In this trial, both brolucizumab groups demonstrated non-inferiority to aflibercept 2 mg in terms of BCVA outcomes at both 48 and 96 weeks [17,18]. At week 48, approximately 60% of patients maintained a 12-week dosing interval. Real-world studies have also shown that brolucizumab effectively resolves exudation and improves BCVA across different AMD subtypes. The reported occlusion rate of polypoidal lesions after the loading phase ranges from 57% to 90% [19,20,21]. Several studies demonstrated that brolucizumab is effective for eyes with neovascular AMD, which poorly respond to aflibercept 2.0 mg. A few studies have reported that brolucizumab can extend the treatment interval by almost 1 month at 12 months or 18 months, although BCVA remains unchanged after switching [22,23].

Faricimab, the second-generation VEGF inhibitor following brolucizumab, is a bispecific antibody that inhibits VEGF-A and angiopoietin 2 with high binding affinity. The TENAYA/LUCERN trials evaluated whether intravitreal administration of faricimab (6.0 mg/0.05 mL) after four consecutive monthly administrations was comparable to bimonthly aflibercept 2.0 mg after three consecutive monthly administrations of aflibercept 2.0 mg using 1329 cohorts at 271 sites worldwide [24]. The BCVA change from the baseline with the faricimab group was non-inferior to the aflibercept 2.0 mg group in both TENAYA/LUCERN (TENAYA: faricimab 5.8 letters vs. aflibercept 2.0 mg, 5.1 letters, LUCERN: faricimab 6.6 letters vs. aflibercept 2.0 mg 6.6 letters). At 48 weeks, the treatment interval greater than 12 weeks in the faricimab group was 79.7% and 77.8% in the TENAYA and LUCERN trials, respectively.

Aflibercept 8 mg is a second-generation VEGF inhibitor developed after brolucizumab and faricimab. The PULSAR trial investigated the efficacy of aflibercept 8 mg in eyes with exudative AMD using 1011 patients. The patients were randomly assigned to the aflibercept 2.0 mg group and the aflibercept 8.0 mg group. The aflibercept 2.0 mg group treatment regimen was the same as the VIEW study. The aflibercept 8.0 mg group was randomly assigned to the 12-week interval or 16-week interval after the induction phase. If the criteria were not met on each visit, the treatment interval was shortened in each group. This study showed that aflibercept 8 mg was comparable to aflibercept 2 mg in terms of BCVA improvement (aflibercept 2.0 mg: +7.6 letters, aflibercept 8.0 mg 12-week interval: +6.7 letters, aflibercept 8.0 mg 16-week interval: +6.2 letters), while offering a longer treatment interval. Regardless of the initial treatment schedule, more than 80% of patients were maintained on 12-week intervals [25].

Despite its potential, there is limited real-world data on the use of aflibercept 8 mg, specifically for the treatment of PCV. In this study, we investigated the short-term efficacy and safety of aflibercept 8 mg for PCV and compared it with brolucizumab.

## 2. Results

A total of 48 eyes from 48 patients with PCV were included in this study, comprising 32 patients treated with brolucizumab and 16 patients treated with aflibercept 8 mg. Baseline demographic and clinical characteristics are summarized in Table 1. There were no significant differences in baseline demographic and clinical characteristics between the two groups.

**Figure 1:**
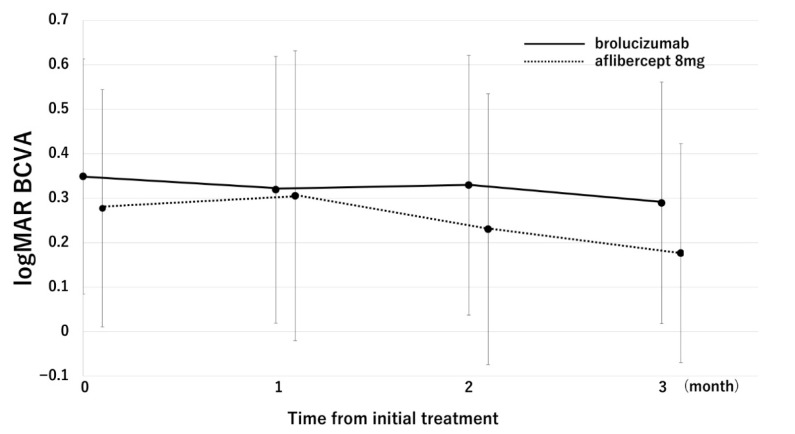
Changes in the BCVA in the aflibercept 8 mg and brolucizumab groups. In the aflibercept 8 mg group, BCVA significantly improved from 0.28 ± 0.27 (baseline) to 0.31 ± 0.33 (1-month visit), 0.23 ± 0.30 (2-month visit), and 0.18 ± 0.25 (3-month visit) (*p* = 0.36, 0.15, and 2.3 × 10^−3^, respectively). In the brolucizumab group, BCVA improved from 0.35 ± 0.26 (baseline) to 0.32 ± 0.30 (1-month visit), 0.33 ± 0.29 (2-month visit), and 0.29 ± 0.27 (3-month visit) (*p* = 0.22, 0.48, and 0.08, respectively).

Figure 1 shows changes in BCVA in the aflibercept 8 mg and brolucizumab groups. In the brolucizumab group, BCVA improved from 0.35 ± 0.26 at baseline to 0.29 ± 0.27 at the 3-month visit, but the change did not reach statistical significance (*p* = 0.08). In contrast, BCVA significantly improved in the aflibercept 8 mg group, from 0.28 ± 0.26 at baseline to 0.18 ± 0.25 at 3 months (*p* = 2.3 × 10^−3^).

Table 2 presents the results of multivariate regression analysis for BCVA at 3 months. Better BCVA outcomes were associated with better baseline BCVA and lower CRT, regardless of the treatment group. There were no significant differences in BCVA at 3 months between the two drugs. In the aflibercept 8 mg group, CRT significantly decreased from 358 ± 152 μm at baseline to 218 ± 87 μm, 200 ± 77 μm, and 192 ± 76 μm at 1, 2, and 3 months, respectively (all *p* < 0.001). SCT also significantly decreased from 186 ± 76 μm at baseline to 161 ± 71 μm, 155 ± 62 μm, and 153 ± 67 μm at 1, 2, and 3 months, respectively (all *p* < 0.001). Similarly, in the brolucizumab group, CRT significantly decreased from 382 ± 157 μm at baseline to 242 ± 129 μm, 211 ± 122 μm, and 198 ± 98 μm at 1, 2, and 3 months, respectively (all *p* < 0.001). SCT significantly decreased from 201 ± 78 μm at baseline to 178± 75 μm, 170 ± 71 μm, and 167± 60 μm at 1, 2, and 3 months, respectively (all *p* < 0.001).

At the 3-month visit, CRT was reduced by 46.3% and 48.4% in the aflibercept 8 mg and brolucizumab groups, respectively (*p* > 0.05). SCT was reduced by 17.7% and 17.4% in the aflibercept 8 mg and brolucizumab groups, respectively (*p* > 0.05).

**Figure 2:**
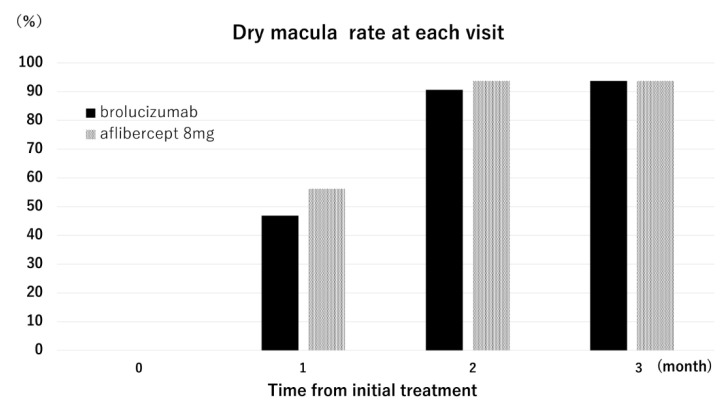
Dry macula rate in the aflibercept 8 mg group and the brolucizumab group. At the 1-month visit, the dry macula rates were 56.3% and 46.9% in the aflibercept 8 mg group and brolucizumab groups, respectively. At the 2-month visit, the dry macular rates were 93.8% and 90.6% in the aflibercept 8 mg group and brolucizumab groups, respectively. At the 3-month visit, the dry macula rate was 93.8% in both groups.

**Figure 3:**
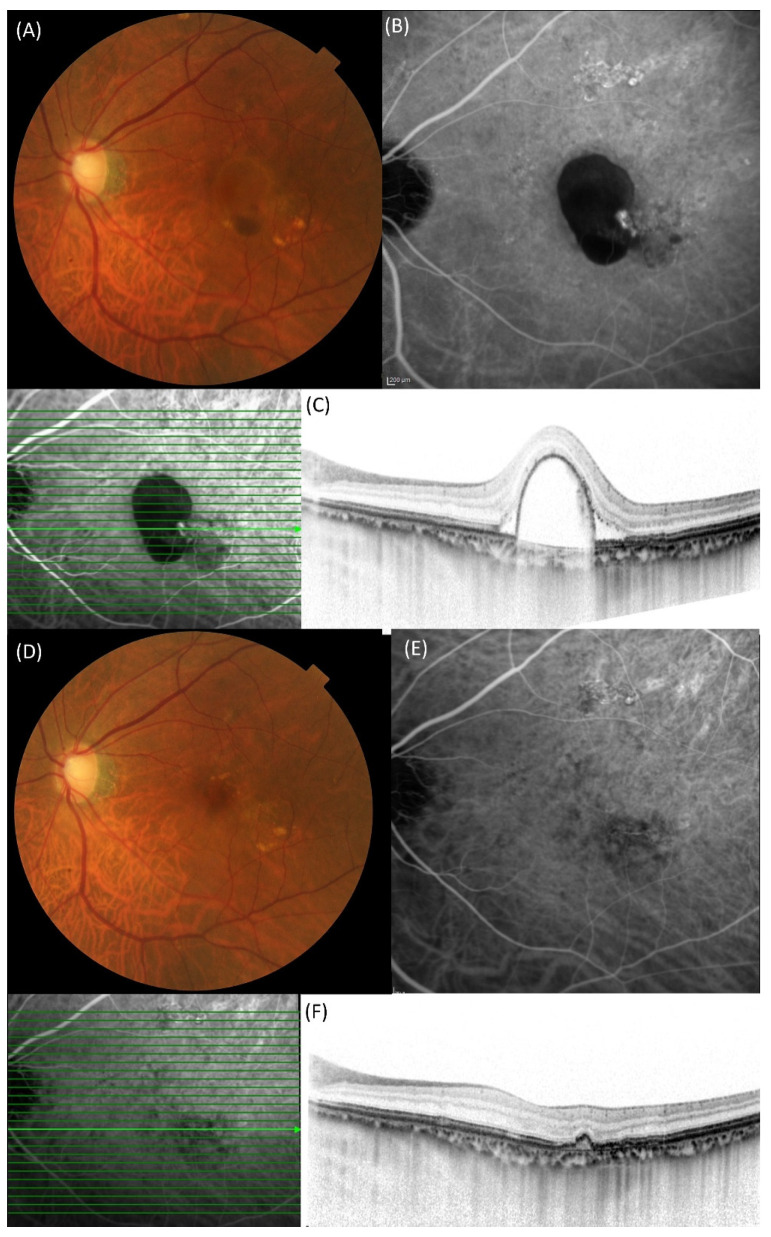
A 76-year-old man presenting with polypoidal choroidal vasculopathy with best-corrected visual acuity of 0.7 (decimal format). (**A**) Fundus photograph showing serous retinal pigment epithelial detachment (PED) with subretinal hemorrhage. (**B**) Indocyanine green angiography (ICGA) demonstrated polypoidal lesions inside PED. (**C**) Horizontal optical coherence tomography (OCT) revealed polypoidal lesions beneath the retinal pigment epithelium (RPE) in a dome-shaped PED, corresponding to hyperfluorescence on ICGA. (**D**) The serous PED and subretinal hemorrhage disappeared on fundus photograph one month after the third injection of aflibercept 8 mg. (**E**) Regression of polypoidal lesions was observed on ICGA one month after the third injection of aflibercept 8 mg. (**F**) Horizontal OCT demonstrated an RPE bump corresponding to the site, with resolution of the polypoidal lesions.

Figure 2 illustrates the dry macula rate at each visit. At 3 months, the dry macula rate was 93.8% in both groups. Complete regression of polypoidal lesions was observed in 62.5% (10/16) and 75.0% (24/32) of eyes in the aflibercept 8 mg and brolucizumab groups, respectively (*p* = 0.57, Chi-square test). A representative case treated with aflibercept 8 mg is shown in Figure 3.

No systemic adverse events were observed in either group. Intraocular inflammation (IOI) was observed in two eyes treated with brolucizumab, but not in any eyes treated with aflibercept 8 mg. Of the two affected eyes, one developed anterior chamber inflammation without posterior segment involvement, while the other developed an arterial occlusion one month after the third injection. This case was described in detail elsewhere [26].

## 3. Discussion

In the early 1990s, PCV was introduced as “idiopathic PCV”, which was predominantly seen in black women [27]. Polypoidal lesions were frequently seen in the macula area in Asians; in contrast, polypoidal lesions were located near the optic disk in the original description [28]. The treatment of PCV was originally photocoagulation, aiming at the disruption of polyps. Since PDT has been clinically available, it has become the standard treatment of PCV [29,30,31,32,33,34,35]. After the advent of ranibizumab, treatment of PCV was shifted to VEGF inhibitor monotherapy or PDT combined with VEGF inhibitor in the real world [36,37]. The EVEREST study investigated the effect of verteporfin PDT combined with ranibizumab or alone versus ranibizumab monotherapy in patients with symptomatic PCV using a total of 61 Asian patients and demonstrated that the combination therapy involving PDT and ranibizumab is superior to ranibizumab monotherapy in terms of complete regression of polypoidal lesions, with no significant differences in BCVA improvement (PDT with ranibizumab group: 10.9 ± 10.9 letters vs. ranibizumab monotherapy: 9.2 ± 12.4 letters) [38]. The PLANET study investigated whether intravitreal administration of aflibercept 2 mg is effective for eyes with PCV and compared aflibercept 2 mg monotherapy with aflibercept 2 mg and rescue PDT (active PDT) [39]. In this randomized clinical trial, 318 patients were included, and patients were randomly assigned to either the aflibercept 2 mg monotherapy group or the aflibercept 2 mg plus rescue PDT group after undergoing induction therapy consisting of three consecutive 4-weekly administrations of aflibercept 2 mg. At 52 weeks, the BCVA improvement was comparable in both treatment groups (+10.8 letters vs. +10.7 letters), with a similar reduction in CRT. In this study, the benefit of adding PDT was not confirmed. The discrepancy regarding the usefulness of PDT between these two randomized clinical trials might be due to the difference in the VEGF inhibitor and/or the difference in the timing of applying PDT.

Since the advent of second-generation VEGF inhibitors in 2020, the treatment of exudative AMD has shifted from first-generation agents, such as ranibizumab and aflibercept 2 mg, to second-generation agents, including brolucizumab and faricimab. These newer drugs offer the advantage of extended treatment intervals with a similar BCVA improvement, potentially reducing the burden on both patients and physicians. Among them, brolucizumab may exert the strongest anti-exudative effect due to its small molecular weight, which allows for high-dose formulation in a single injection while maintaining solubility and stability. However, focal adverse events—particularly IOI—remain a significant concern following brolucizumab administration [40].

In the present study, we investigated the short-term outcomes of aflibercept 8 mg, the most recently introduced second-generation VEGF inhibitor, in eyes with polypoidal choroidal vasculopathy (PCV) and compared the results with those of brolucizumab. BCVA significantly improved in the aflibercept 8 mg group, from 0.28 ± 0.27 to 0.18 ± 0.25, and the dry macula rate at 3 months was 93.8% in both groups. While the PULSAR study did not report a 3-month dry macula rate, it reported a rate of 62–65% at 16 weeks, regardless of the treatment regimen [25]. These findings suggest that PCV may be more responsive to aflibercept 8 mg.

The mean age of the PULSAR and the HAWK/HARRIER trials is 74.6 ± 8.2 and 76.5 ± 8.7 years, respectively. The mean age of brolucizumab 6 mg and aflibercept 8 mg in the study is 75.4 ± 8.0 (range: 61–89) and 73.9 ± 9.2 (range: 58–89) years, respectively. Although the demographic factor, including age, is a potential associated factor of responsiveness to treatment, the impact of age on visual outcomes may be similar to that observed in previous studies.

In terms of CRT reduction, aflibercept 8 mg achieved a 46–48% decrease, similar to that observed with brolucizumab. Both drugs showed comparable anatomical responses during the induction phase of three consecutive monthly administrations. In the PULSAR trial, CRT decreased by 150 μm from 371 ± 128 at 12 weeks. In the present study, CRT decreased by 166 μm from 358 ± 152 at 3 months. Change in CRT was not different between studies. Although aflibercept affects the suppression of placental growth factor and VEGF-B, it is unclear how these affect the anatomical response, including CRT and SCT. Further investigation is needed to evaluate potential differences in anatomical responses during the maintenance phase.

Previous studies have reported that complete regression of polypoidal lesions during the induction phase may positively impact long-term visual outcomes and extend the time to first recurrence and reduce the number of recurrences [41,42]. Sayanagi et al. demonstrated that eyes achieving complete regression showed significantly improved BCVA at 12 months and experienced fewer recurrences compared with those without complete regression [41]. Therefore, selecting an appropriate VEGF inhibitor capable of regressing polypoidal lesions is essential. In our study, the complete regression rate was higher in the brolucizumab group (75.0%) than in the aflibercept 8 mg group (62.5%), although this difference was not statistically significant. This might be due to the small number of cohorts. Further studies with larger cohorts are warranted to determine which agent is more effective for inducing polyp regression.

Regarding adverse events, no systemic adverse events were reported in either group. Regarding focal adverse events, IOI occurred in two eyes (6.3%) treated with brolucizumab, whereas no cases were observed in the aflibercept 8 mg group. This difference was not statistically significant. Several case reports have demonstrated that IOI due to aflibercept 8 mg is characterized by mild anterior chamber inflammation, with or without vitritis, and without retinal arteriole occlusion. In the recent case series reporting IOI due to faricimab, the presentation frequently seen was anterior chamber inflammation and vitritis, and retinal vascular occlusion was not seen [43,44]. IOI due to aflibercept 8 mg might lead to a low risk of severe visual deterioration if adequate treatment is performed [45]. Several studies have identified factors associated with IOI following brolucizumab administration [46,47,48]; however, the mechanism developing IOI has not been fully understood [16]. In the present study, one patient developed vascular occlusion without vision loss. However, vascular occlusion is potentially vision-threatening. Therefore, the decision regarding the drug profile should be carefully made, and further consideration is needed. Also, careful attention should be paid to intraocular adverse events following administration of aflibercept 8 mg [49,50].

This study has several limitations, including its retrospective design and single-center setting. The second limitation is that PCV has multiple variations in phenotypes, including the presence or absence of pachychoroid phenotypes, with or without a branching vascular network. The third is that the drug selection is time-dependent, which may cause selection bias. Further multicenter, prospective studies with larger sample sizes are necessary to determine the superiority of VEGF inhibitors in terms of polypoidal lesion regression and BCVA improvement.

In summary, aflibercept 8 mg is effective in improving BCVA and resolving exudation during the induction phase in eyes with PCV. It is comparable to brolucizumab in terms of dry macula rate, anatomical response, and the complete regression of polypoidal lesions.

## 4. Materials and Methods

A retrospective medical chart review was conducted on eyes with PCV that received three consecutive monthly administrations of either brolucizumab (6.0 mg/mL; 0.05 mL) or aflibercept 8.0 mg (114.3 mg/mL; 0.07 mL). The selection of drugs was time-dependent: brolucizumab was administered between August 2020 and December 2023, while aflibercept 8.0 mg was administered between June 2024 and April 2025.

This study was approved by the Institutional Review Board of the University of Yamanashi (approval code: no. 2892, approval date: 17 February 2025) and was conducted in accordance with the tenets of the Declaration of Helsinki. Written informed consent was obtained from all patients prior to the initiation of treatment.

At the initial visit, all patients underwent a comprehensive ophthalmic examination, including BCVA measurement using a Landolt chart, intraocular pressure measurement, slit-lamp examination with or without a 78-diopter lens, color fundus photography, spectral-domain optical coherence tomography (SD-OCT) using Spectralis (Heidelberg Engineering, Dossenheim, Germany), and fluorescein and indocyanine green angiography (FA/ICGA) using the HRA 2 confocal laser scanning system (Heidelberg Engineering, Dossenheim, Germany). SD-OCT scans were regularly performed using 25 horizontal B-scans covering a 20° × 25° area of the macula. If polypoidal lesions were located between scan lines, the scanning pattern was adjusted at the physician’s discretion, as we previously described [19].

The inclusion criterion was treatment-naïve eyes with PCV, excluding those that had undergone any prior treatments except cataract surgery. Exclusion criteria included (1) the presence of other macular diseases aside from PCV and (2) a prior history of vitrectomy, PDT, or other anti-VEGF treatments.

PCV was diagnosed based on ICGA and simultaneously performed SD-OCT. On ICGA, PCV was defined by the presence of hyperfluorescent polypoidal lesions, and on SD-OCT, by retinal pigment epithelium (RPE) protrusions or dome-shaped RPE detachments.

All patients were followed monthly up to the 3-month visit and received intravitreal injections of either brolucizumab or aflibercept 8.0 mg at baseline, 1 month, and 2 months. At the 1-month and 2-month visits, patients underwent examinations identical to those at baseline, excluding FA/ICGA. At the 3-month visit, a comprehensive examination including FA/ICGA was performed to confirm the presence or absence of polypoidal lesion regression.

Central retinal thickness (CRT) and subfoveal choroidal thickness (SCT) were measured at each visit. CRT was defined as the vertical distance between Bruch’s membrane and the inner limiting membrane at the fovea. SCT was defined as the vertical distance between the choroidoscleral border and Bruch’s membrane at the fovea.

## 5. Statistical Analysis

Statistical analyses were conducted using SPSS software (version 12, IBM, Tokyo, Japan). The Chi-square test or Mann–Whitney U test was used to test for differences in categorical or continuous variables. Differences in continuous variables between baseline and after treatment were evaluated using a paired *t*-test. A *p*-value less than 0.05 is statistically significant.

## Figures and Tables

**Table 1:** Baseline demographic and clinical characteristics of patients with polypoidal choroidal vasculopathy between the brolucizumab and aflibercept 8 mg group.

	Brolucizumab (*n* = 32)	Aflibercept 8 mg (*n* = 16)	*p*-Value
Age (years)	75.40 ± 8.0	73.9 ± 9.2	0.61
Male	24 (75%)	11 (68.8%)	0.91
Mean baseline log MAR BCVA	0.35 ± 0.26	0.28 ± 0.27	0.40
Baseline central retinal thickness (μm)	382.0 ± 157.1	358.2 ± 152.2	0.55
Baseline subfoveal choroidal thickness (μm)	201.5 ± 77.9	185.9 ± 76.1	0.55
Number of polyps	2 ± 1.22	1.4 ± 0.6	0.083
Maximum polyp size (μm)	378.1 ± 236.3	434.4 ± 154.1	0.082

**Table 2:** Multivariate analysis of factors associated with best-corrected visual acuity (log MAR unit) at 3 months after treatment.

Variables	β-Coefficient	*p*-Value
Age (years)	−0.03	0.74
Type of treatment (0: aflibercept 8 mg, 1: brolucizumab)	0.099	0.27
Baseline log MAR BCVA	0.81	2.1 × 10^−10^
Baseline subfoveal choroidal thickness (μm)	−0.027	0.77
Baseline central retinal thickness (μm)	0.24	0.013
Number of polyps	−0.09	0.35
Maximum polyp size (μm)	−0.06	0.53

## Data Availability

Data is contained in the paper.

## Abbreviations

AMD	age-related macular degeneration
BCVA	best-corrected visual acuity
CRT	central retinal thickness
ICGA	indocyanine green angiography
IOI	intraocular inflammation
MNV	macular neovascularization
PCV	polypoidal choroidal vasculopathy
PDT	photodynamic therapy
SCT	subfoveal choroidal thickness
VEGF	vascular endothelial growth factor
